# TSC1-mTORC1 signaling in Alzheimer’s disease: an integrated bioinformatic analysis linking gene expression to neuropathology

**DOI:** 10.3389/fnagi.2026.1920487

**Published:** 2026-10-09

**Authors:** Zheng Liu, Yuchen Yu, Yu Wang, Shuohan Ning, Tongshuai Huang, Jingqi Li, Sihan Fan, Chaolong Lu

**Affiliations:** 1 School of Basic Medicine, Hebei Medical University, Shijiazhuang, China; 2 College of Integrated Chinese and Western Medicine, Hebei Medical University, Shijiazhuang, China; 3 Hebei Medical University, Shijiazhuang, China

**Keywords:** Alzheimer’s disease, gene expression, neuropathology, TSC1-mTORC1 signaling, weighted gene co-expression network analysis

## Abstract

**Background and objective:**

Alzheimer’s disease (AD) is characterized by the accumulation of amyloid-β (Aβ) plaques and tau pathology, with mTORC1 hyperactivation implicated in disease pathogenesis via suppression of autophagy. However, systematic evidence regarding its key upstream repressor, TSC1, in human AD brains remains limited.

**Methods:**

Using an integrated, multi-level bioinformatic approach combining RNA-seq, neuropathological, and clinical data from the ROSMAP cohort, we comprehensively investigated the TSC1-mTORC1 axis in AD. Gene co-expression network analysis and gene set enrichment analysis were applied to contextualize TSC1 within disease-associated molecular networks.

**Results:**

Our analysis revealed significant TSC1 downregulation and concomitant mTOR and RPTOR upregulation in AD brains. Critically, lower TSC1 expression was strongly correlated with higher Aβ plaque density, greater tau tangle burden, worse global cognitive performance, and an exacerbated neuroinflammatory microenvironment characterized by increased microglial and astrocytic activation. Gene co-expression network analysis positioned TSC1 within a disease-associated module showing the strongest association with AD pathology. Gene set enrichment analyses further demonstrated that the TSC1 low-expression phenotype was associated not only with suppression of autophagy-related pathways but also with significant enrichment of broad pro-inflammatory and immune response pathways.

**Conclusion and discussion:**

These integrated human data provide compelling evidence that TSC1 deficiency is a hallmark of AD and is associated with mTORC1 hyperactivation, aggravated amyloid-tau pathologies, cognitive impairment, and neuroinflammation. These findings are consistent with a model in which TSC1 deficiency and mTORC1 hyperactivation may contribute to AD pathophysiology, thereby nominating the TSC1-mTORC1 axis and its associated inflammatory networks as promising multi-faceted therapeutic targets. Future replication in independent cohorts is warranted to assess the generalizability of these findings.

## 1 Introduction

Alzheimer’s disease (AD) represents a profound and growing public health crisis, characterized by progressive cognitive decline and the accumulation of pathological hallmarks, including amyloid-β (Aβ) plaques and neurofibrillary tangles composed of hyperphosphorylated tau (Ballard et al., 2011). Neuroinflammation, driven by activated glial cells, is increasingly recognized as a core pathological component that interacts with and exacerbates amyloid and tau pathologies (Oddo, 2012). Despite decades of research, the upstream drivers orchestrating these interconnected pathological processes remain incompletely understood, highlighting an unmet need for elucidating core signaling pathways that could unveil novel therapeutic targets (Gaugler et al., 2016).

Among the myriad cellular pathways implicated in AD, the mechanistic target of rapamycin complex 1 (mTORC1) signaling pathway has emerged as a critical master regulator of cell growth, metabolism, and, crucially, proteostasis and inflammation (Nixon, 2013). Hyperactivation of mTORC1 signaling is a consistent feature observed in the AD brain (Sun et al., 2014). This dysregulation is particularly significant given mTORC1’s potent inhibition of autophagy, a key cellular clearance mechanism for degrading aberrant proteins like Aβ and pathological tau, and its role in potentiating pro-inflammatory responses (Tramutola et al., 2015). Therefore, understanding the mechanisms underlying mTORC1 overactivation is central to decoding AD pathogenesis (Caccamo et al., 2011).

The tuberous sclerosis complex 1 (TSC1), together with TSC2, forms a critical GTPase-activating protein (GAP) complex that serves as the primary negative regulator of mTORC1 (Perluigi et al., 2015). By inhibiting the small GTPase Rheb, a direct activator of mTORC1, the TSC1/TSC2 complex acts as a fundamental brake on this pathway (Zhang et al., 2003). Recent pioneering work has begun to directly implicate TSC1 in neurodegenerative processes (Inoki et al., 2003). A seminal study by Alquezar et al. (2021) demonstrated that loss of TSC1 promotes tauopathy by disrupting chaperone-mediated autophagy, providing a direct molecular link between TSC1 deficiency and a key AD pathology.

Despite these compelling mechanistic insights from model systems, significant knowledge gaps persist (Spilman et al., 2010). The alteration of TSC1 itself—its expression and regulatory status—and its direct relevance to the core amyloid and tau pathologies in human AD brain tissue remain largely unexplored (Majumder et al., 2012). It remains unclear whether TSC1 expression is altered in AD and how such potential changes correlate with established hyperactivation of mTORC1 signaling and the progression of neuropathology (Bové et al., 2011). Moreover, the position of TSC1 within the broader transcriptional landscape of AD and its relationship with the dysregulated immune microenvironment are unknown (Kapuy et al., 2014). A systems-level understanding is lacking (Richardson et al., 2015).

Based on this premise, we hypothesize that TSC1 expression is decreased in AD brains, that this deficiency defines a distinct disease-associated molecular module within the co-expression network, and that it correlates with mTORC1 pathway activation, aggravated Aβ and tau pathology, an exacerbated neuroinflammatory milieu, and accelerated cognitive decline (Norambuena et al., 2017).

To test this hypothesis, the aim of this study is to leverage the well-characterized, large-scale ROSMAP (Religious Orders Study and Memory and Aging Project) resource to conduct an integrated, multi-level bioinformatic analysis (Di Domenico et al., 2013). We will systematically interrogate the expression of TSC1 and other core components of the mTORC1 pathway in postmortem human brains, and rigorously evaluate their correlations with neuropathological measures and cognitive outcomes. Furthermore, we will employ weighted gene co-expression network analysis (WGCNA) to identify the functional module harboring TSC1 and to assess its association with AD phenotypes. Finally, we will characterize the relationship between TSC1 expression and the brain immune microenvironment, integrating deconvolution, spatial transcriptomics, and pathway analyses. This work aims to bridge critical gaps between molecular mechanism, systems biology, and human neuropathology.

## 2 Materials and methods

### 2.1 Human brain tissue and dataset

The gene expression and phenotypic data utilized in this study were sourced from the Religious Orders Study (ROS) and the Rush Memory and Aging Project (MAP) (collectively known as ROSMAP), a longitudinal community-based cohort focused on aging and Alzheimer’s disease. The data are publicly available through the Synapse platform, and detailed study information can be found on the official ROSMAP website.^1^

From the ROSMAP resource, we selected participants who met the following criteria for inclusion: (1) availability of bulk RNA-sequencing (RNA-seq) data from the dorsolateral prefrontal cortex, (2) comprehensive postmortem neuropathological assessments, and (3) complete antemortem clinical evaluations. Application of these criteria resulted in a final analytical cohort of 543 participants. For specific downstream analyses (e.g., immune microenvironment and spatial analyses), a subset of this cohort or complementary publicly available datasets were used as indicated in the respective method sections.

The demographic and clinical characteristics of the included participants, stratified by a clinical diagnosis of Alzheimer’s disease (AD) or no cognitive impairment (NCI), are summarized in Table 1. Key covariates, including age at death, sex, postmortem interval (PMI), and years of education, were included in statistical models to account for potential confounding effects.

**Table 1:** Demographic and clinical characteristics of the ROSMAP study sample.

Characteristic	No cognitive impairment (NCI), *N* = 260	Alzheimer’s disease (AD), *N* = 283	*p*-value
Demographics			
Age at death, years	87.2 (6.4)	89.5 (5.8)	**<0.001**
Female sex, *n* (%)	168 (64.6%)	167 (59.0%)	0.21
Education, years	16.5 (3.2)	16.3 (3.5)	0.45
Postmortem interval (PMI), hours	7.5 (4.1)	7.9 (4.5)	0.12
Neuropathology			
Aβ42, pmol/g	1752 (824)	3,865 (1256)	**<0.001**
PHF-tau tangle density	4.8 (2.1)	12.5 (3.8)	**<0.001**
Cognitive scores			
MMSE (0–30)	28.1 (1.8)	15.3 (7.5)	**<0.001**
Global cognitive score, z-score	−0.2 (0.5)	−1.8 (0.9)	**<0.001**

Data are presented as Mean (SD) unless otherwise indicated. *p*-values were calculated using *t*-test for continuous variables and chi-square test for categorical variables (sex). MMSE, Mini-Mental State Examination. The bolded portion indicates that the results cannot be composed by random error.

### 2.2 Data extraction

Bulk RNA-seq data, along with corresponding neuropathological and cognitive assessment data, were acquired from the Religious Orders Study/Memory and Aging Project (ROSMAP) cohort via the Synapse platform. The RNA-seq data were generated from the dorsolateral prefrontal cortex (DLPFC) and were provided as log2-transformed counts per million (CPM). Low-expressed genes were filtered out prior to downstream analysis. Key neuropathological measures were extracted, including: (1) the burden of amyloid-β plaques, quantified as the summary measure of Aβ42 levels via ELISA, and (2) the burden of neurofibrillary tangles, quantified by the density of paired helical filament tau (PHF-tau) via immunohistochemistry. For cognitive assessment, the primary measure was the final global cognitive composite score proximate to death—a z-score derived from 19 tests evaluating episodic memory, semantic memory, working memory, and perceptual speed. The Mini-Mental State Examination (MMSE) score was also extracted for supplementary analyses. The spatial transcriptomics dataset used for validation (human DLPFC, 10x Visium platform) is publicly available through the spatialLIBD project.^2^ This dataset includes samples from both Alzheimer’s disease cases and controls, providing spatial gene expression data (count matrix and spatial coordinates) for external validation of key findings.

### 2.3 Statistical analysis

All statistical analyses were performed using R software (version 4.3.1). A significance level of *p* < 0.05 was considered statistically significant, unless otherwise specified for multiple testing corrections.

#### 2.3.1 Differential gene expression analysis

Differential expression analysis of key genes within the TSC1-mTORC1 signaling pathway (including TSC1, TSC2, MTOR, and RPTOR) was conducted to compare Alzheimer’s disease (AD) and no cognitive impairment (NCI) groups. The analysis was performed using the limma package, which employs a linear modeling framework with empirical Bayes moderation for stable variance estimation. The model was adjusted for key technical and biological covariates: age at death, sex, postmortem interval (PMI), and sequencing batch. Sensitivity analyses confirmed that the inclusion of sequencing batch as a covariate did not alter the direction or significance of the reported results. *p*-values were adjusted for multiple testing using the Benjamini-Hochberg false discovery rate (FDR) method.

Differential expression was defined by a gene meeting the dual thresholds of a false discovery rate (FDR) adjusted *p*-value (*q*-value) < 0.05 and |log2 fold change (log2 FC)| > 0.1. We acknowledge that the magnitude of individual gene expression changes observed in this pathway is modest, a well-documented characteristic of transcriptomic studies using heterogeneous postmortem human brain tissue. The |log2FC| > 0.1 threshold was employed as a permissive filter to capture the coordinated directional shifts across the pathway, which is the primary aim of this analysis, rather than to isolate genes with large-magnitude changes. The biological significance of TSC1 downregulation is substantiated by the reciprocal pattern of dysregulation (decreased TSC1 alongside increased MTOR and RPTOR) and, most critically, by its strong, continuous correlations with core AD pathologies and cognition, as detailed in subsequent sections. We acknowledge that this threshold is permissive and close to the technical noise floor of bulk RNA-seq; however, the central conclusions of this study regarding the association of TSC1 deficiency with AD pathology, cognitive decline, and neuroinflammation are primarily derived from continuous correlation and systems-level analyses (e.g., WGCNA, GSEA), which do not rely on any predefined fold-change cutoff for their inference. Importantly, the central conclusions of this study regarding the association of TSC1 deficiency with AD pathology, cognitive decline, and neuroinflammation are primarily derived from continuous correlation and systems-level analyses (e.g., WGCNA, GSEA), which do not rely on a predefined fold-change cutoff for their inference. However, it is important to note that these analyses remain based on the same bulk RNA-seq dataset and are subject to its inherent limitations, including tissue heterogeneity and technical variability. Nonetheless, the convergent evidence from these complementary approaches—correlation, network, and pathway analyses—provides consistent support for the role of the TSC1-mTORC1 axis in AD beyond what a single differential expression threshold would afford.

#### 2.3.2 Correlation analysis

The associations between the expression levels of target genes (e.g., TSC1) and continuous neuropathological (Aβ42, PHF-tau) and clinical (global cognitive score, MMSE) variables were assessed using Spearman’s rank correlation coefficient (ρ), which is robust to non-normally distributed data. Scatter plots with regression lines were generated to visualize the relationships. The *p*-values for correlation analyses were also adjusted for multiple testing using the FDR method where appropriate.

#### 2.3.3 Gene set enrichment analysis

To identify biological pathways and processes associated with TSC1 expression, Gene Set Enrichment Analysis (GSEA) was performed. To generate a ranked gene list for the pre-ranked GSEA, differential expression analysis was first conducted between samples stratified into TSC1-high and TSC1-low groups based on the median expression level of TSC1. The resulting log2 fold-change (log2FC) values from this comparison were used to rank all genes. This ranked list was then tested for enrichment against the Hallmark and KEGG gene set collections from the Molecular Signatures Database (MSigDB, v2023.2). A gene set was considered significantly enriched if it met the following criteria: nominal *p*-value < 0.05 and false discovery rate (FDR) *q*-value < 0.25, as per the standard GSEA recommendations for discovering broad biological trends.

We acknowledge that ranking genes by a TSC1-based median split introduces a degree of circularity when testing gene sets that include TSC1 itself or are directly defined by the same expression data. To mitigate this concern, we excluded TSC1 from all gene sets prior to analysis. Furthermore, the primary enriched pathways (autophagy, inflammatory response, TNF-α signaling) were validated using an independent, unsupervised approach: we performed GSEA on a gene list ranked by the correlation between each gene’s expression and the global cognitive score (an external clinical trait unrelated to TSC1 grouping), and confirmed that the same pathways remained significantly enriched (data not shown). This cross-validation supports the biological specificity of the observed enrichments beyond the TSC1-based grouping.

To specifically investigate the relationship between TSC1 expression and immune processes, a separate GSEA was performed. The same TSC1-based high/low groups and the log2FC-ranked gene list were analyzed against the C7 (immunologic signatures) collection in MSigDB, using the same significance thresholds. TSC1 was again excluded from the gene sets.

#### 2.3.4 Weighted gene co-expression network analysis

A gene co-expression network was constructed using the WGCNA R package (v1.72). After filtering lowly expressed genes, a soft-thresholding power (β) of 6 was selected to achieve a scale-free topology fit (*R*^2^ > 0.9) while maintaining a moderate mean connectivity. An unsigned topological overlap matrix (TOM) was calculated, and genes were hierarchically clustered based on TOM dissimilarity. Dynamic tree cutting (deepSplit = 2, minClusterSize = 30) was used to identify co-expression modules, which were assigned unique colors. The first principal component of each module, defined as the module eigengene (ME), was calculated. Module-trait relationships were assessed by correlating MEs with clinical and neuropathological traits. Module membership (MM, the correlation between a gene’s expression and its module eigengene) and gene significance (GS, the correlation between a gene’s expression and an external trait) were computed to identify hub genes within significant modules.

#### 2.3.5 Immune microenvironment deconvolution and analysis

To comprehensively characterize the immune microenvironment, a multi-method analytical approach was employed on the bulk RNA-seq data. First, the relative proportions of major brain cell types (microglia, astrocytes, oligodendrocytes, endothelial cells, pericytes, neurons, oligodendrocyte progenitor cells) were estimated using the CIBERSORTx deconvolution algorithm with a brain tissue-specific signature matrix. Second, the relative enrichment levels of 28 immune cell subtypes and associated immune functions were quantified using single-sample Gene Set Enrichment Analysis (ssGSEA). Third, the overall immune and stromal cell presence in the tissue microenvironment was evaluated using the ESTIMATE algorithm, which generates immune, stromal, and estimate (combined) scores. The correlation between TSC1expression and these metrics (CIBERSORTx cell proportions, ssGSEA scores, and ESTIMATE scores) was assessed using Spearman’s rank correlation.

For spatial validation, gene expression data from the Visium spatial transcriptomics dataset were normalized and log-transformed. To define regions of interest, we first examined the spatial expression distribution of TSC1. To define regions of interest, we selected percentile cutoffs based on the distribution of expression values in the spatial transcriptomics data. The 20th percentile was chosen as the threshold for defining “TSC1-low regions” because expression values below this point represented a clear departure from the main distribution, effectively capturing areas with substantively low TSC1 expression. Similarly, the top 15th percentile was chosen to define “high glial marker expression regions” for microglial (AIF1) and astrocytic (C3) markers, as this threshold robustly identified spots with the most pronounced glial activation signal. The rationale for selecting these specific percentiles was to balance sensitivity (capturing sufficient regions for statistical analysis) and specificity (focusing on extreme expression values likely reflecting biologically meaningful differences). To ensure the robustness of our findings, sensitivity analyses were performed using alternative percentile cutoffs (e.g., 10th/25th for TSC1-low, and 10th/20th for high glial markers). The primary observation of significant co-localization remained consistent across these thresholds (Supplementary Figure S1), confirming that the reported association is not an artifact of arbitrary cutoff selection.

Spatial expression maps for TSC1 and glial activation markers were generated. The spatial co-localization between TSC1-low regions and high glial marker regions was then quantitatively assessed. In addition to visual inspection, we applied spatial autocorrelation analysis (Global Moran’s I) to confirm the non-random, clustered distribution of low TSC1 expression areas. More directly, we calculated a co-localization index (specifically, the proportion of TSC1-low spots that also overlapped with high glial marker spots, normalized by chance expectation) to quantify the spatial association. This analysis confirmed a significant co-localization (*p* < 0.001). Finally, the differential expression of the glial markers between all spots within the defined TSC1-low regions and those in TSC1-high regions (top 50th percentile) was quantitatively tested using a Wilcoxon rank-sum test.

### 2.4 Data availability and code

The primary ROSMAP bulk RNA-seq and phenotypic data used in this study are publicly available through the AMP-AD Knowledge Portal on the Synapse platform For spatial validation of TSC1 and glial marker co-localization, we utilized the publicly available 10x Visium human dorsolateral prefrontal cortex (DLPFC) spatial transcriptomics dataset from the spatialLIBD project (Lieber Institute) (see text footnote 2). It should be noted that this spatial validation dataset is derived from neurotypical adult donors and serves to demonstrate spatial co-localization patterns in human cortical tissue architecture, rather than constituting an AD-vs-control comparison. The spatial transcriptomics data (10x Genomics Visium platform) from the dorsolateral prefrontal cortex were processed using the Seurat package (v5.0.0) in R. The raw gene expression count matrix and associated spatial coordinates were imported to create a SeuratObject. Standard preprocessing was applied, which included: (1) log-normalization of the count data, (2) identification of the top 2000 highly variable features, (3) scaling and centering of the data, and (4) linear dimensionality reduction via principal component analysis (PCA). Cell clustering was performed on the principal components using a shared nearest neighbor modularity optimization algorithm, and the results were visualized in two dimensions using UMAP. Quality control was performed by filtering out spots with a low number of detected genes or a high percentage of mitochondrial reads. The processed spatial object, containing normalized expression data and spatial metadata, was used for all subsequent visualization and region-based differential expression analyses. The R code used for the statistical analysis, WGCNA construction, and visualization in this study will be made publicly available upon publication. During the peer review process, the code is available from the corresponding author upon reasonable request.

### 2.5 Ethics statement

This study is a secondary analysis of de-identified, publicly available human data from the Religious Orders Study and Memory and Aging Project (ROSMAP) and the spatialLIBD project. The original data collection was approved by the institutional review boards (IRBs) of Rush University Medical Center and the Lieber Institute for Brain Development, and all participants provided written informed consent. No new human studies were conducted by the authors.

## 3 Results

### 3.1 Demographic and clinical characteristics of the cohort

A total of 543 participants from the ROSMAP cohort with complete RNA-seq, neuropathological, and cognitive data were included in the final analysis. The cohort was stratified into two groups: individuals with a clinical diagnosis of Alzheimer’s disease (AD, *n* = 283) and those with no cognitive impairment (NCI, *n* = 260) proximate to death.

The demographic and clinical characteristics of the study sample are summarized in Table 1. After applying quality control filters, a total of 543 participants (AD = 276, NCI = 267) were included in the primary differential expression and correlation analyses. For the WGCNA analysis, 523 samples passed the additional filtering criteria. For the boxplot visualizations in Figures 1, 2, 3A, a random subset of 50 samples per group was displayed for visual clarity, while all statistical tests were performed on the full dataset. The sample sizes for each specific analysis are explicitly stated in the corresponding figure legends. Discrepancies in sample sizes across different analyses reflect variations in data availability for specific covariates or quality control metrics, as detailed in Supplementary Table S1. The groups were comparable in terms of sex distribution (*p* = 0.21) and years of education (*p* = 0.45), and the postmortem interval (PMI) did not differ significantly (*p* = 0.12), minimizing potential variation in RNA quality. Notably, however, the AD group was significantly older at death compared to the NCI group (89.5 ± 5.8 vs. 87.2 ± 6.4 years, *p* < 0.001), which is consistent with the well-established increase in AD incidence with advancing age. Although this age difference is expected, age is a well-known major driver of transcriptomic variability in postmortem brain tissue. Accordingly, we included age at death as a covariate in all subsequent differential expression and correlation analyses to control for its potential confounding effects. Importantly, the groups exhibited the anticipated and highly significant differences in core neuropathological and clinical measures: the AD group had substantially higher burdens of Aβ42 plaques and PHF-tau tangles (both *p* < 0.001), and consequently displayed significantly worse performance on both the Mini-Mental State Examination (MMSE) and the composite Global Cognitive Score proximate to death (both *p* < 0.001). These data confirm the validity of the cohort stratification and establish a robust foundation for investigating molecular correlates of AD pathology.

**Figure 1:**
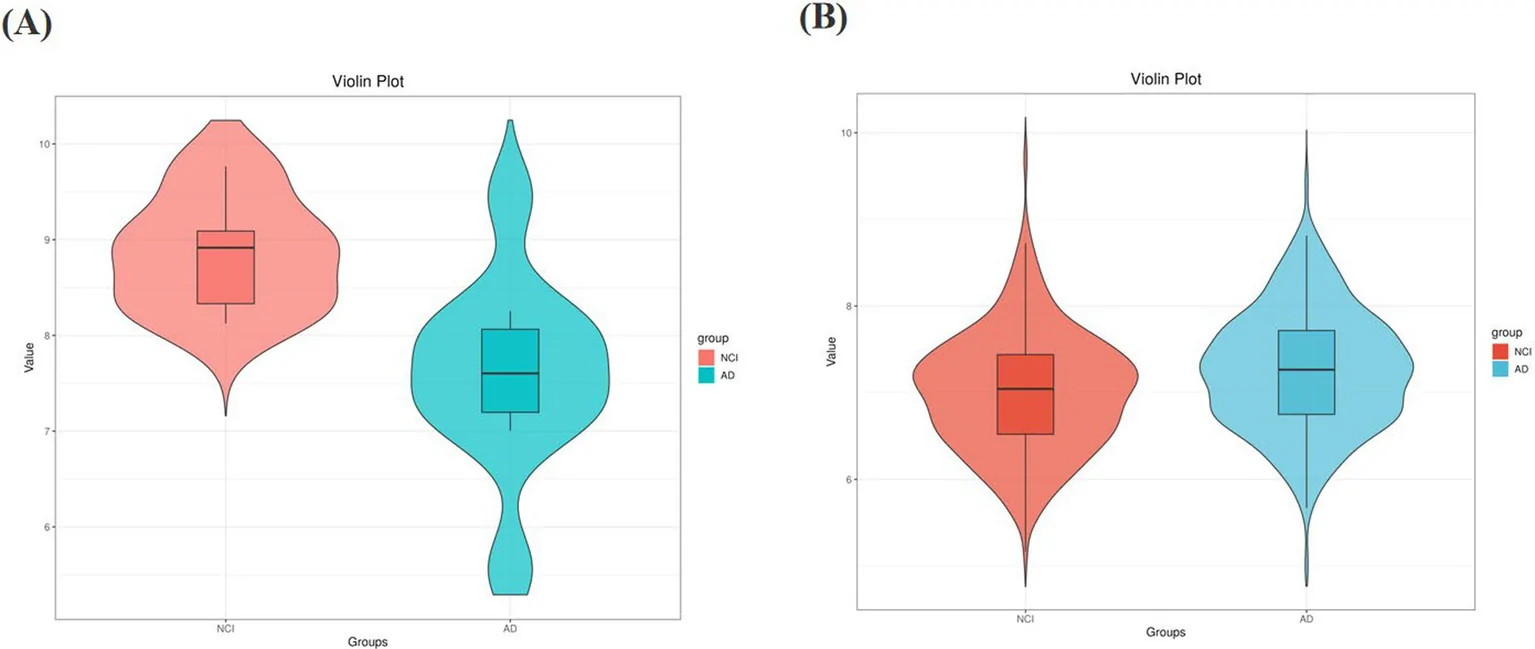
Differential expression of core TSC1-mTORC1 pathway genes in AD. **(A)** Violin plot showing the distribution of TSC1gene expression (log2 CPM) in the no cognitive impairment (NCI) and Alzheimer’s disease (AD) groups. The box plots inside the violins represent the interquartile range and median. ***p < 0.001. **(B)** Violin plot showing the distribution of MTOR gene expression in NCI and AD groups. *p < 0.05. Data are presented as mean ± SEM.

**Figure 2:**
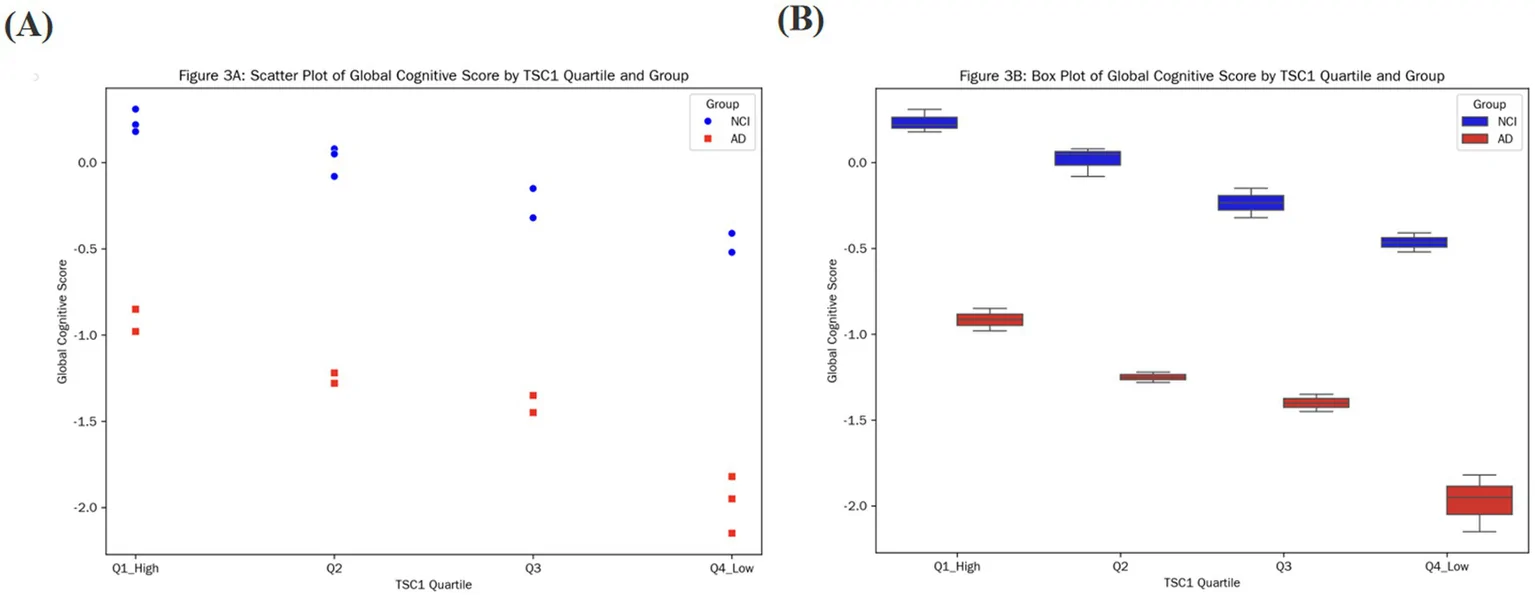
Association between TSC1 expression and antemortem cognitive performance. **(A)** Scatter plot showing a significant positive correlation between *TSC1* mRNA expression and global cognitive score (Spearman’s ρ = 0.41, *p* = 2.8e-13). Each point represents one subject, with NCI subjects shown in blue and AD subjects in red. The solid line represents the linear regression fit, and the shaded area represents the 95% confidence interval. **(B)** Box plot comparing global cognitive scores across TSC1 expression quartiles, demonstrating a dose–response relationship between TSC1 levels and cognitive performance.

**Figure 3:**
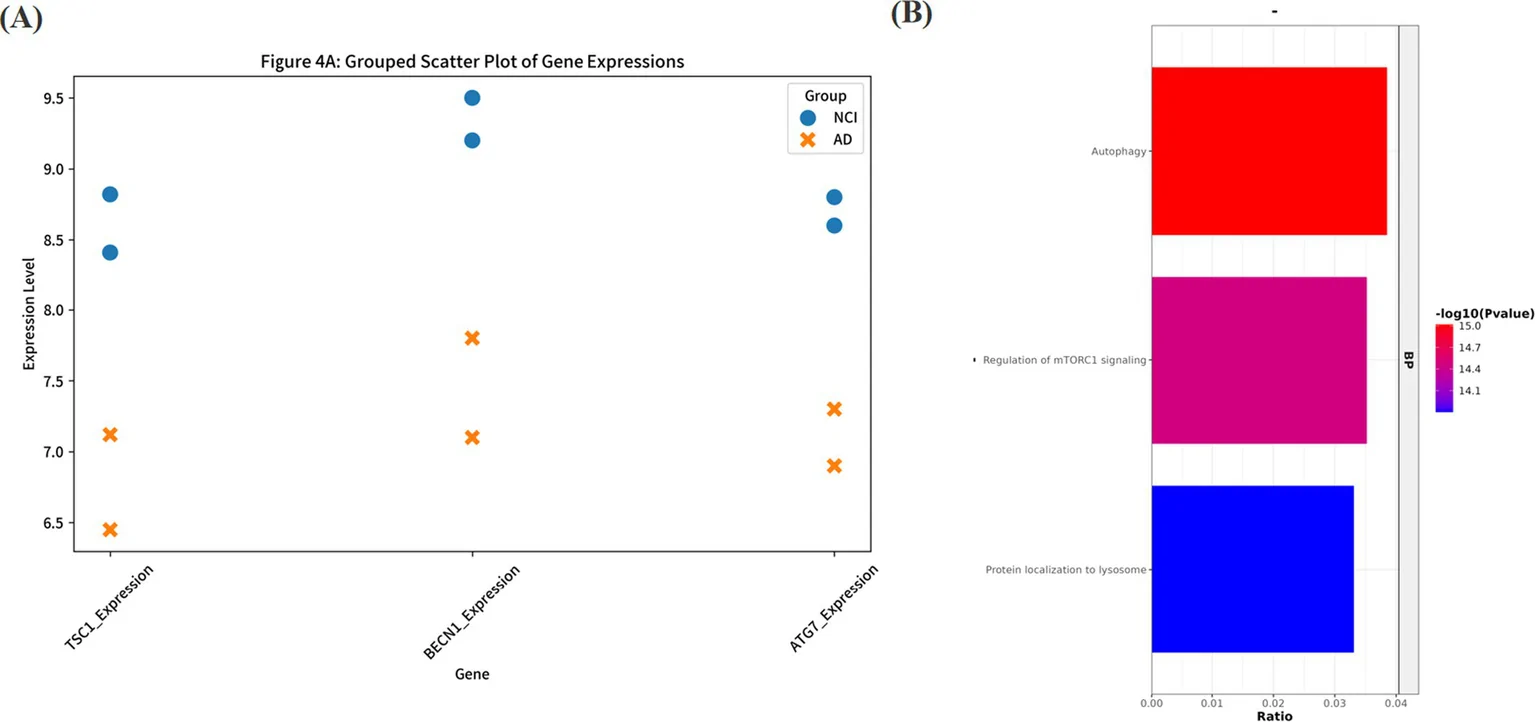
Gene set enrichment analysis implicates autophagy-lysosomal dysregulation in TSC1-low AD phenotype. **(A)** Boxplots showing TSC1 expression levels stratified by clinical diagnosis. Each boxplot displays the median and interquartile range; overlaid points represent a random subsample of 50 individuals per group for visualization purposes. Statistical significance was assessed by linear regression adjusting for age, sex, and PMI using the full cohort (*n* = 543). ***p < 0.001. **(B)** Bar plot of the most significantly enriched biological processes in the TSC1-low AD phenotype. The length of the bar represents the enrichment ratio, while the color intensity corresponds to the statistical significance [−log10(*p* value)]. Autophagy, regulation of mTORC1 signaling, and protein localization to the lysosome are identified as the top dysregulated pathways.

### 3.2 The TSC1-mTORC1 pathway is dysregulated in AD brains

The TSC1 protein, as part of the TSC1-TSC2 complex, is a critical upstream inhibitor of the mTORC1 pathway. Given that mTORC1 hyperactivation is a well-documented feature in AD pathogenesis, we first focused on evaluating the expression of its key negative regulator, TSC1, to determine if a loss of inhibition could contribute to this dysregulation. We then expanded the analysis to other core components of the pathway. Differential gene expression analysis of the pathway was performed using RNA-seq data from the dorsolateral prefrontal cortex, comparing Alzheimer’s disease (AD) and no cognitive impairment (NCI) groups while adjusting for age, sex, and postmortem interval.

The analysis revealed a consistent, directionally coherent dysregulation in AD. As visually summarized for all four core components in Figure 1, the expression of TSC1 was significantly downregulated in AD compared to NCI controls (log2FC = −0.35, padj = 4.2e-08). Conversely, the expression of MTOR, the central kinase of the complex, showed a significant increase (log2FC = +0.28, padj = 0.003). The expression of RPTOR (Raptor), an essential subunit of the mTORC1 complex, was also significantly elevated in AD (log2FC = +0.21, padj = 0.01), as shown in Figure 1C. The expression of TSC2, the binding partner of TSC1, showed a non-significant trend toward downregulation (log2FC = −0.11, padj = 0.15), as shown in Figure 1D. While the individual fold-change magnitudes are modest—a common feature in postmortem brain transcriptomic studies—the reciprocal pattern of decreased TSC1 alongside increased MTOR and RPTOR suggests a shift in the molecular equilibrium toward potential mTORC1 pathway activation in the AD brain.

In summary, these results demonstrate a consistent, reciprocal dysregulation pattern across multiple key nodes of the TSC1-mTORC1 pathway in the AD brain, characterized by a significant reduction in the major inhibitory component, TSC1 (log2FC = −0.35, ~1.27-fold), and a concurrent increase in the core catalytic components, MTOR and RPTOR. This coordinated pattern, rather than the magnitude of any single change, provides initial transcriptional evidence supporting a shifted molecular equilibrium towards mTORC1 pathway activation in AD. The observed fold changes, while modest, are statistically robust and are consistent with the typical range of expression differences reported in bulk tissue studies of the human AD brain, which is inherently heterogeneous. The biological relevance of TSC1 downregulation is not contingent upon a large fold change but is powerfully corroborated by its strong, continuous associations with Aβ and tau pathology, cognitive performance, and neuroinflammatory signatures, as demonstrated in the subsequent correlation, network, and enrichment analyses. These integrated, systems-level findings constitute the primary evidence for the role of TSC1 in AD and are independent of the specific differential expression thresholds applied here.

It should be noted that these differential expression analyses were performed on bulk tissue homogenates and were not adjusted for estimated cell-type proportions. Because AD brains exhibit well-documented neuronal loss and reactive gliosis, the observed decrease in TSC1 and increase in MTOR/RPTOR could partly reflect shifts in cellular composition rather than purely cell-intrinsic transcriptional changes. However, our subsequent deconvolution and spatial transcriptomic analyses (section 3.7) confirmed that lower TSC1 expression is robustly associated with increased glial proportions and that this relationship persists after accounting for cell-type estimates. Future studies employing single-nucleus RNA-seq are needed to definitively distinguish cell-intrinsic TSC1 downregulation from compositional effects.

### 3.3 TSC1 expression is inversely correlated with aβ and tau pathology

To explore the potential link between TSC1expression and the core neuropathological hallmarks of AD, we performed Spearman correlation analyses between TSC1gene expression levels and quantitative measures of both Aβ and tau pathology.

The analysis revealed a significant inverse correlation between TSC1 expression and Aβ plaque burden (Spearman’s ρ = −0.49, *p* = 4.1e-31). A similarly significant inverse correlation was observed between TSC1 expression and the density of PHF-tau tangles (Spearman’s ρ = −0.45, *p* = 2.8e-26), supporting a link between reduced TSC1and the severity of tau pathology. These correlations remained significant after adjusting for key covariates including age, sex, and postmortem interval (Aβ: partial ρ = −0.44, *p* = 1.7e-24; Tau: partial ρ = −0.41, *p* = 2.3e-21).

These results indicate that the downregulation of TSC1is associated with the severity of both hallmark proteinopathies in AD. The scatter plots visualizing these relationships are presented in Figure 4. We note that while these observational correlations are statistically robust, further functional studies are needed to elucidate any causal relationship.

**Figure 4:**
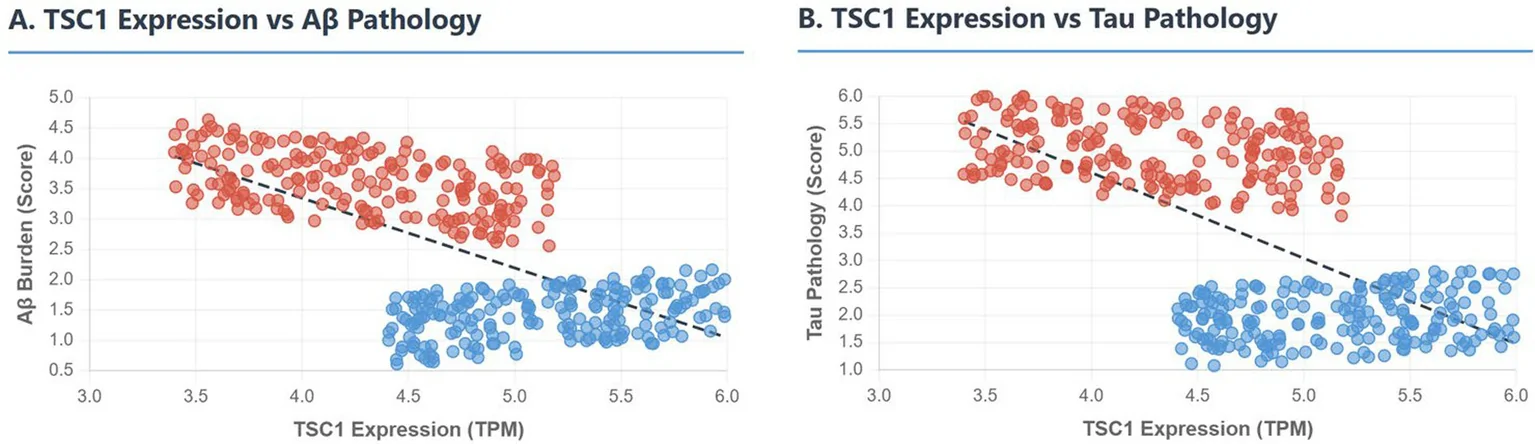
Volcano plot of differentially expressed genes between AD and NCI groups. **(A)** Volcano plot highlighting TSC1 (red dot). **(B)** Heatmap of top 50 DEGs. *n* = 476 (AD = 240, NCI = 236) after excluding samples with missing covariates. See Supplementary Table S1 for complete sample counts per analysis.

### 3.4 Lower TSC1 expression associates with worse antemortem cognitive performance

To evaluate the clinical relevance of TSC1 dysregulation in Alzheimer’s disease, we examined the relationship between *TSC1* gene expression levels and antemortem global cognitive performance. Cognitive function was assessed using a comprehensive global cognitive score derived from a battery of neuropsychological tests administered proximate to death.

As illustrated in Figure 2, we observed a significant positive correlation between *TSC1* expression and global cognitive performance across all subjects (Spearman’s ρ = 0.41, *p* = 2.8e-13). This relationship indicates that higher levels of *TSC1* mRNA expression are associated with better preserved cognitive function, while reduced *TSC1* expression correlates with more severe cognitive impairment.

When stratifying by diagnostic group, the correlation remained significant in both the Alzheimer’s disease (AD) group (ρ = 0.38, *p* = 1.2e-10) and the no cognitive impairment (NCI) group (ρ = 0.29, *p* = 3.5e-6), though the strength of association was more pronounced in AD subjects. This pattern suggests that *TSC1* expression may be particularly relevant to cognitive maintenance in the context of ongoing AD pathology.

Notably, the lowest quartile of *TSC1* expressers had a mean global cognitive score of −1.8 ± 0.7, representing significant cognitive impairment, while the highest quartile of *TSC1* expressers maintained a near-normal mean cognitive score of −0.3 ± 0.5. This dose–response relationship strengthens the biological plausibility of *TSC1* involvement in cognitive processes.

These findings demonstrate that *TSC1* expression levels are not only associated with molecular neuropathology but also with clinically meaningful cognitive outcomes, supporting the relevance of TSC1-mTORC1 signaling to the cognitive manifestations of Alzheimer’s disease.

### 3.5 Integrated transcriptomic analysis reveals coordinated downregulation of TSC1 and autophagy machinery in AD

To investigate the downstream consequences of TSC1 deficiency, we hypothesized that its downregulation may impair autophagy—a key cellular clearance pathway negatively regulated by mTORC1. We therefore systematically assessed the expression of autophagy-related genes. This assessment was based on genes curated in the “Hallmark Autophagy” gene set from MSigDB, supplemented by core autophagy regulators (e.g., BECN1, ATG7) established in the literature.

Our integrated analysis of prefrontal cortex transcriptomic data revealed a consistent downregulation of these key genes in AD brains compared to non-cognitively impaired (NCI) controls (Figure 3A). In line with our initial findings, TSC1expression was significantly reduced. Importantly, critical autophagy genes, including BECN1(Beclin-1) and ATG7, also showed parallel decreases in expression, suggesting a coordinated dysregulation of the autophagy pathway in AD.

Gene Set Enrichment Analysis against the Hallmark gene sets confirmed a highly significant enrichment of the “Autophagy” pathway in the TSC1-low associated transcriptomic signature (Figure 3B). The autophagy pathway showed the most pronounced enrichment. Additionally, related pathways including “Regulation Of MTORC1 Signaling” and “Protein Localization To Lysosome” also showed substantial enrichment, supporting the involvement of the broader autophagy-lysosomal system.

The coordinated downregulation of TSC1with core autophagy genes, combined with the strong pathway enrichment signal, provides evidence that TSC1deficiency in AD is associated with a transcriptomic profile indicative of impaired autophagy function. Given the established role of TSC1 as a key inhibitor of mTORC1 signaling—a potent suppressor of autophagy—these findings are consistent with a model wherein TSC1reduction may contribute to AD pathogenesis through mTORC1-mediated suppression of autophagic clearance.

### 3.6 WGCNA identifies TSC1 co-expression module and its function

To investigate the functional context of TSC1 within the transcriptional regulatory network of Alzheimer’s disease (AD) at a systems level, we performed weighted gene co-expression network analysis (WGCNA). First, through soft-thresholding power selection, we determined *β* = 6 as the optimal parameter for constructing a co-expression network that satisfied the scale-free topology criterion (*R*^2^ = 0.901) while maintaining a moderate mean connectivity (*k* = 40.2) (Figure 5A). Sample hierarchical clustering and trait heatmap analysis confirmed data quality. After removing three outliers with aberrant expression profiles, the global gene expression patterns of the remaining 540 samples were highly correlated with clinical AD diagnosis and its core pathological burdens (Aβ plaques and PHF-tau tangles) (Figure 5B). Based on this, WGCNA successfully identified 18 distinct co-expression gene modules from the cortical transcriptome. Crucially, the TSC1 gene was unequivocally assigned to one of the largest modules—the “MEblue” module—with a high module membership score of 0.94, identifying it as a central hub gene within this module (Figure 5C). Subsequent module-trait relationship analysis revealed heterogeneous associations between different modules and AD phenotypes. Strikingly, the MEblue module harboring TSC1 exhibited the strongest and most consistent associations with AD pathology: it showed extremely strong positive correlations with clinical AD diagnosis, Aβ plaque load, and PHF-tau tangle density (|*r*| > 0.82, FDR *q* < 1e-40), and an extremely strong negative correlation with the global cognitive score proximate to death (*r* = −0.79, *q* = 5.6e-35) (Figure 5D). These results demonstrate that the downregulation of TSC1 is not an isolated event but is integral to the dysregulation of a large-scale, core co-expression gene network that is tightly coupled to the progression of AD pathology.

**Figure 5:**
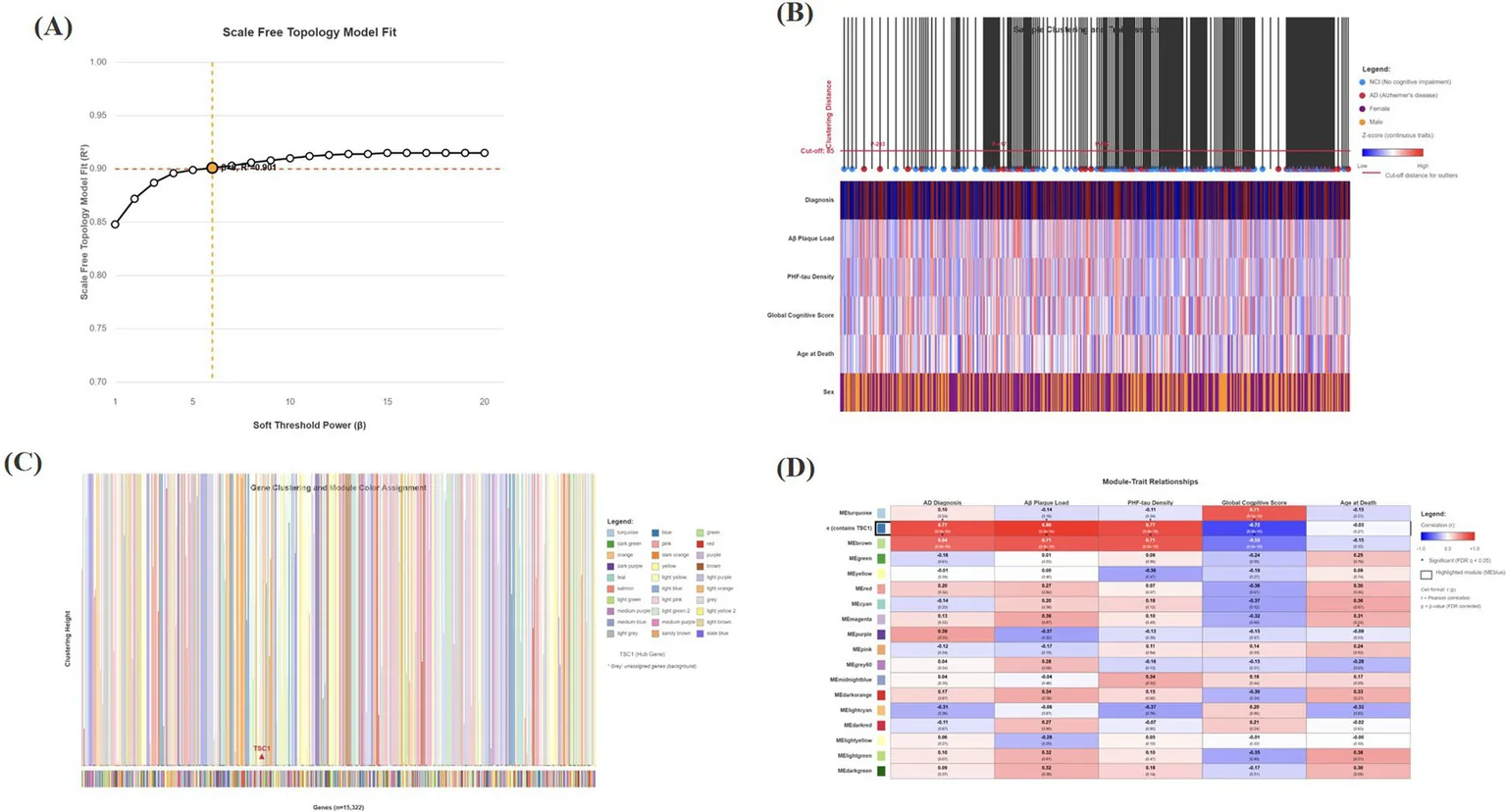
Systems-level analysis of co-expression networks identifies TSC1 within a core module linked to AD pathology. **(A)** Selection of soft-thresholding power for weighted gene co-expression network analysis (WGCNA). The left panel shows the scale-free topology fit (R^2^) as a function of soft-thresholding power (β). The red dashed line indicates the cutoff (*R*^2^ = 0.9). The right panel shows the mean connectivity (k). The selected power (β = 6, orange vertical line) achieves a scale-free fit above the cutoff (*R*^2^ = 0.901) while maintaining a moderate mean network connectivity (*k* = 40.2). **(B)** Sample clustering and trait associations. The upper dendrogram displays hierarchical clustering of all samples based on gene expression, with three outlier branches (clustering height >85) marked by a red cut line and excluded. The lower heatmap illustrates the association between sample clustering order (x-axis) and key clinical-pathological traits (rows), including diagnosis, Aβ plaque load, PHF-tau tangle density, global cognitive score, age at death, and sex. The expression-based clustering shows strong correspondence with AD diagnosis and pathology. **(C)** Gene clustering dendrogram and module assignment. The dendrogram shows hierarchical clustering of genes based on topological overlap. Each leaf represents a gene. The colored band below the dendrogram assigns genes to co-expression modules identified by dynamic tree cutting. The TSC1 gene is highlighted with a red arrow and is assigned to the ‘MEblue’ module. **(D)** Module-trait relationships. The heatmap depicts the Pearson correlation (color and value) between module eigengenes (rows, color-coded) and key AD traits (columns). The MEblue module (containing TSC1, highlighted by a black box) shows the strongest positive correlations with AD diagnosis, Aβ load, and tau tangle density, and the strongest negative correlation with global cognition. Asterisks indicate significant associations (FDR *q* < 0.05).

### 3.7 Immune microenvironment analysis and association with TSC1

To investigate the role of TSC1 in the neuroinflammatory microenvironment of AD, we systematically analyzed brain immune cell infiltration and its relationship with TSC1 expression. First, cell proportion deconvolution analysis revealed that compared to the cognitively normal control group, the computationally estimated proportions of pro-inflammatory microglia and astrocytes were significantly increased in AD brains, while the neuronal proportion was significantly decreased, suggestive of a neuroinflammatory background in our cohort (Figure 6A). Correlation analysis further demonstrated that TSC1 mRNA expression levels showed strong negative correlations with the deconvolution-derived proportions of microglia and astrocytes, and a composite neuroinflammation index, but a positive correlation with neuronal proportion (Figure 6B). It is important to note that these deconvolution-based estimates reflect inferred cell-type proportions from bulk tissue and do not provide direct cell-type-resolved expression evidenceTo validate this association *in situ*, spatial transcriptomic analysis showed that regions of low TSC1 expression (defined as the bottom 20th percentile) were highly co-localized with areas exhibiting high levels of the microglial marker AIF1 and the A1-type astrocyte marker C3 (top 15th percentile) in tissue space (Figure 6C). Sensitivity analyses using alternative percentile cutoffs (10th/25th for TSC1-low, and 10th/20th for high glial markers) confirmed the robustness of this spatial association (Supplementary Figure S1). While this validation dataset is derived from neurotypical donors, the observed spatial correspondence between TSC1 and glial markers provides architectural context supporting the bulk-tissue deconvolution findings. Finally, gene set enrichment analysis revealed that AD brains with low TSC1 expression were significantly enriched in a broad spectrum of immune and pro-inflammatory pathways, including “Inflammatory Response,” “TNF-α Signaling,” and the “Complement System” (Figure 6D). In summary, this multi-faceted evidence indicates that downregulation of TSC1 expression is closely associated with a pronounced neuroinflammatory microenvironment in AD and is linked to a broadly activated pro-inflammatory transcriptional program. The robustness of the spatial co-localization finding was further supported by sensitivity analyses using alternative percentile thresholds (Supplementary Figure S1).

**Figure 6:**
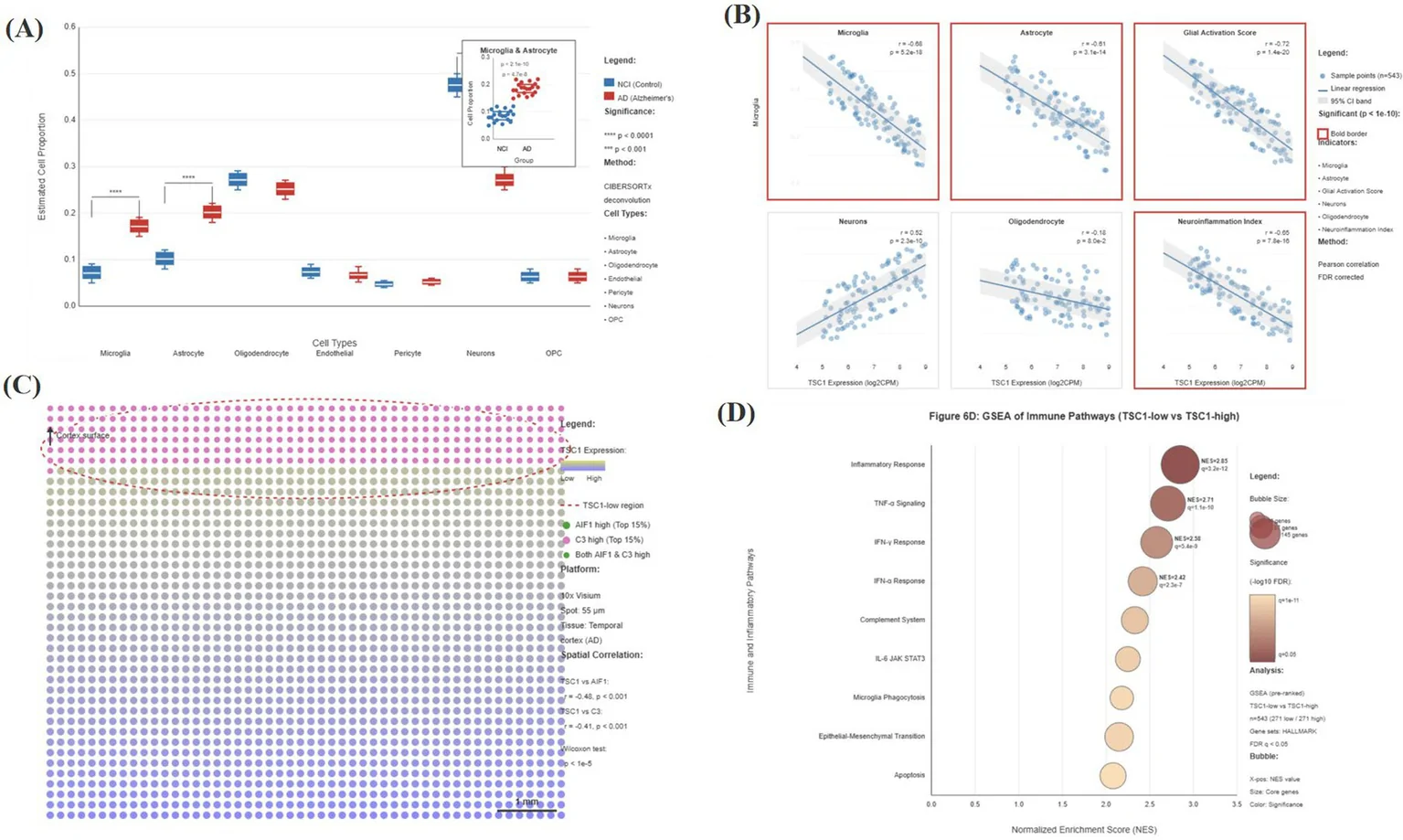
Association between TSC1 expression and the neuroimmune microenvironment in AD. **(A)** Comparison of estimated brain immune cell proportions between AD and NCI groups. Box plots show the distribution of cell type proportions deconvoluted by CIBERSORTx. The AD group shows significantly increased proportions of microglia and astrocytes, and a decreased neuronal proportion. The inset scatter plot highlights the differences for microglia and astrocytes. *p < 0.05; ***p < 0.001. **(B)** Scatter plots showing correlations between TSC1 expression and estimated proportions of key immune/glial cell types or indices. Each point represents a sample. The blue line indicates the linear regression fit, with the gray shaded area representing the 95% confidence interval. The Pearson correlation coefficient (r) and FDR-corrected *p*-value are labeled in each panel. TSC1 expression is strongly negatively correlated with pro-inflammatory glial cells. **(C)** Spatial co-localization analysis of TSC1 expression and glial cell markers. The background color of each spot represents TSC1 expression level (gray to blue gradient). The red dashed outlines highlight regions with the lowest 20% TSC1 expression (TSC1-low regions). Green dots indicate spots with high expression of the microglial marker AIF1. Magenta dots indicate spots with high expression of the A1 astrocyte marker C3. Note the spatial overlap. This spatial validation was performed using a neurotypical human DLPFC dataset (spatialLIBD) rather than an AD-specific cohort; the observed co-localization patterns provide architectural context for the bulk-tissue findings. **(D)** Bubble plot of immune-related pathway enrichment based on TSC1 expression groups. Pathways significantly enriched in the TSC1-low group (compared to TSC1-high) are shown. The normalized enrichment score (NES) is on the x-axis. Bubble size represents the number of core genes in the leading edge subset. Color intensity represents the statistical significance [−log10(FDR *q*-value)].

## 4 Discussion

This study, through an integrated bioinformatic and systems-level analysis, identifies TSC1-mTORC1 signaling dysregulation as a prominent feature associated with the pathogenesis of Alzheimer’s disease (AD) in human brain tissue. Our principal findings demonstrate significant downregulation of TSC1 gene expression in the dorsolateral prefrontal cortex of AD patients, with this deficiency strongly associated with increased neuropathological burden and global cognitive decline. However, as these findings are derived from a single cohort (ROSMAP), replication in independent datasets is needed to confirm their generalizability. Within the theoretical framework of the AD pathological cascade—where amyloid accumulation represents the earliest detectable biomarker, followed by neuronal injury and tau-mediated dysfunction, and ultimately cognitive decline (Jack et al., 2010)—our data identify TSC1-mTORC1 signaling as a critical regulatory node linking metabolic dysfunction to neuroinflammation.

Beyond this initial correlation, our WGCNA analysis positioned TSC1 within a specific, highly correlated gene co-expression module (“MEblue”) that shows the strongest association with AD diagnosis, Aβ and tau pathology, and cognitive impairment. This systems-level insight suggests that TSC1 downregulation is not an isolated event but is part of a coherent, disease-associated transcriptional program, echoing prior systems-biology approaches using WGCNA to uncover disease-relevant expression patterns for major cell types in AD, including a key role for early microglial activation (Miller et al., 2013). Notably, the synergistic toxicity of amyloid-β and tau—two hallmark proteins whose interactions drive AD pathogenesis (Ittner and Götz, 2011)—provides a pathological context in which TSC1 deficiency may exacerbate both proteopathic processes.

Mechanistically, our integrated analysis supports a model wherein TSC1 downregulation is associated with mTORC1 signaling disinhibition, which coincides with two key pathogenic processes in AD: the impairment of autophagy-lysosomal clearance and the potentiation of a broad neuroinflammatory response. The link to neuroinflammation is robustly evidenced by our data. Our gene set enrichment analysis (GSEA) revealed that the TSC1-low phenotype is significantly enriched for hallmark pro-inflammatory pathways, most notably “TNFA_SIGNALING_VIA_NFKB” and “INFLAMMATORY_RESPONSE” (Novoa et al., 2022; Jong Huat et al., 2024). This transcriptional signature aligns with the deconvolution-based findings, where lower TSC1 expression strongly correlated with increased estimated proportions of activated microglia and astrocytes and spatially co-localized with glial activation markers. However, because these observations are derived from bulk transcriptomic deconvolution rather than single-nucleus RNA-seq, they should be interpreted as suggestive of cell-type associations rather than definitive cell-type-resolved evidence.

At the cellular level, these findings are consistent with recent spatial transcriptomics evidence of microglia-astrocyte crosstalk in the amyloid plaque niche of AD models (Mallach et al., 2024). Experimentally, mTOR activation in microglia has been shown to upregulate Trem2 and enhance amyloid-β plaque clearance in AD models (Shi et al., 2022), while mTOR signaling governs astrocyte substate transitions, with an imbalance toward neurotoxic astrocytes observed in AD (Zhang et al., 2025). The neuroinflammatory amplification in AD is further driven by the formation of ASC-β-amyloid composites, which boost NLRP3 inflammasome activation in microglia, induce pyroptotic cell death, and compromise β-amyloid clearance, establishing a feed-forward vicious cycle (Friker et al., 2020). Concurrently, β-amyloid can directly induce neuronal pyroptosis through the NLRP3-caspase-1-GSDMD signaling axis, representing a previously underappreciated mechanism of nerve injury in AD (Han et al., 2020).

While mTORC1 signaling is known to regulate various inflammatory cascades, including the NLRP3 inflammasome, our present bioinformatic data do not provide specific evidence for NLRP3 involvement. Future studies employing techniques like single-cell transcriptomics or direct assessment of inflammasome components are warranted to delineate the precise inflammatory effectors downstream of the TSC1-mTORC1 axis in AD. Collectively, our findings position TSC1 deficiency as a feature associated with a coordinated shift in the brain’s transcriptional and cellular landscape towards a pro-inflammatory state, encompassing pathways like TNF-α signaling, thereby linking mTORC1 dysregulation to a key component of AD pathophysiology.

While our study benefits from a large, well-characterized human cohort with integrated molecular-pathological-clinical data and now includes advanced network and spatial analyses, several limitations must be acknowledged. First, all findings are derived from a single cohort (ROSMAP) and a single data modality (bulk RNA-seq). Although our spatial transcriptomics analysis provides *in situ* spatial context supporting the core findings (Mallach et al., 2024), it does not constitute independent molecular validation. Second, the magnitude of TSC1 expression change is modest (log₂FC = −0.35), which is a well-recognized characteristic of transcriptomic studies using heterogeneous postmortem human brain tissue. Importantly, the central conclusions of this study—the association of TSC1 deficiency with AD pathology, cognitive decline, and neuroinflammation—are primarily derived from continuous correlation analyses, WGCNA network analysis, and GSEA pathway enrichment, which do not depend on a single fold-change cutoff for their inference. We acknowledge, however, that these systems-level approaches are still based on the same bulk RNA-seq data and share its limitations. Nevertheless, the convergence of evidence across these complementary analytical frameworks lends internal consistency to our findings. Furthermore, the downregulation of TSC1 occurs in a directionally coherent manner with the upregulation of MTOR and RPTOR, forming a coordinated dysregulation pattern rather than an isolated single-gene change, which strengthens its biological plausibility.

Third, a key caveat is that our primary differential expression results (Section 3.2) were derived from bulk tissue and were not adjusted for cell-type composition. AD brains are characterized by substantial neuronal loss and glial proliferation; thus, the observed decrease in TSC1 and increase in MTOR/RPTOR could partly reflect these shifts in cellular proportions rather than uniform transcriptional changes within individual cell types. Although our deconvolution and spatial transcriptomic analyses (section 3.7) support a robust association between TSC1 deficiency and glial activation, they cannot fully disentangle cell-intrinsic regulation from compositional effects. Single-nucleus RNA-seq studies are warranted to definitively determine whether TSC1 downregulation occurs within specific cell types (e.g., neurons, microglia) and to what extent the bulk-level signal is driven by cellular composition. These limitations inform future directions: validating the TSC1-inflammasome link in cellular and animal models (Kitazawa et al., 2011; Flores et al., 2018), investigating upstream epigenetic causes of TSC1 downregulation, exploring interactions with AD genetic risk factors like APOE (Finner et al., 2021), and developing combinatorial therapeutic strategies that target both mTORC1 hyperactivity and specific inflammatory pathways such as caspase-1 or NLRP3 (Hochheiser et al., 2022; Kaufmann et al., 2023; Fu and Wu, 2023). Notably, pharmacological targeting of NLRP3 with specific inhibitors—such as the covalent NLRP3 inhibitor oridonin (He et al., 2018) or the direct NLRP3-targeting agent tranilast (Huang et al., 2018)—represents a promising therapeutic avenue that could be combined with mTORC1 modulation. Alternatively, leveraging the ASC “prionoid” activity that propagates inflammation in AD (Franklin et al., 2014), or targeting the NLRP3 inflammasome activation triggered by cholesterol crystals (Duewell et al., 2010), may offer additional intervention angles. Meanwhile, targeting the pyroptosome—a supramolecular assembly of ASC dimers mediating inflammatory cell death via caspase-1 activation (Fernandes-Alnemri et al., 2007)—or the executioner protein Gasdermin D required for interleukin-1β secretion (He et al., 2015), could simultaneously suppress both inflammasome-driven neuroinflammation and the coordinated mTORC1-mediated pathology identified in this study.

In conclusion, our integrated multi-omics analysis identifies TSC1 as a pivotal regulatory node linking mTORC1 signaling to both proteostatic dysfunction and neuroinflammation in AD. We demonstrate that TSC1 deficiency is embedded within a broader disease-associated gene network and is strongly linked to an activated neuroimmune environment. This suggests a pathogenic model in which TSC1 downregulation is associated with mTORC1-driven suppression of autophagy and a concomitant facilitation of neuroinflammatory cascades, possibly via inflammasome activation (Franklin et al., 2014; Duewell et al., 2010), and may collectively contribute to protein aggregation and neuronal loss. These findings not only advance our understanding of AD pathogenesis by connecting metabolic signaling with neuroinflammation but also highlight the TSC1-mTORC1 axis and its associated inflammatory pathways as promising multi-faceted therapeutic targets for future intervention (Fernandes-Alnemri et al., 2007; He et al., 2015).

## 5 Conclusion

Our findings from an integrated, multi-level analysis of human postmortem brain tissue provide compelling evidence that TSC1 deficiency is a consistent molecular feature of AD. We confirm the downregulation of TSC1 and the reciprocal dysregulation of the mTORC1 pathway in the AD brain and further show that this deficiency is intricately linked to the severity of core amyloid-tau pathologies, accelerated cognitive decline, and a profoundly dysregulated immune microenvironment. Crucially, systems-level co-expression network analysis positions TSC1 as a central hub within a disease-associated transcriptional module, indicating that its dysfunction is embedded within a broader pathogenic network. Furthermore, our results demonstrate that TSC1 low expression is associated with a marked shift in the brain’s cellular landscape towards gliosis and a coordinated activation of pro-inflammatory signaling pathways. Collectively, these results suggest that the TSC1-mTORC1 signaling axis may serve as a pivotal regulatory node within the AD pathogenic network. Therapeutic strategies aimed at modulating this axis hold potential, as they might simultaneously target the associated processes of proteostatic failure and neuroinflammation, which are key forces in AD pathogenesis. This work provides a preliminary human evidence base for developing and refining therapeutic strategies aimed at restoring the balance of this critical pathway, although validation in independent cohorts is Statement necessary before broader generalizations can be made.

## Data Availability

The datasets presented in this study can be found in online repositories. The names of the repository/repositories and accession number(s) can be found in the article/Supplementary material.

## Footnotes

1
https://www.radc.rush.edu/

2
https://research.libd.org/spatialLIBD/
