# Supplementary material for: TSC1-mTORC1 signaling in Alzheimer’s disease: an integrated bioinformatic analysis linking gene expression to neuropathology
Source: Front Aging Neurosci. 2026 Oct 9;18:1920487. doi: 10.3389/fnagi.2026.1920487 (PMC13690809; doi:10.3389/fnagi.2026.1920487)
Supplement: Supplementary file 1 [file Supplementary_file_1.docx]

| **Analysis** | **Total** | **AD** | **NCI** | **Notes** |
| --- | --- | --- | --- | --- |
| Demographic and clinical characteristics (Table 1) | 543 | 276 | 267 | Full cohort after quality control |
| Differential expression analysis (Figs 1, 3, 4A) | 543 | 276 | 267 | All samples passing QC |
| WGCNA network analysis | 523 | 266 | 257 | After removing outlier samples |
| Volcano plot and heatmap (Fig 2) | 476 | 240 | 236 | After excluding samples with missing covariates |
| Boxplot visualizations (Figs 1, 3, 4A) | 543 | 276 | 267 | Statistics computed on full dataset; plots show random subsample of 50 per group |

**Supplementary Materials**

**Supplementary Table S1. Sample sizes for each analysis**

**Supplementary Figure S1. Sensitivity analysis of spatial co-localization using alternative percentile cutoffs.**

The bar plot shows the co-localization index (observed/expected overlap proportion) for TSC1-low regions versus high glial marker regions under different threshold combinations. Error bars represent 95% confidence intervals from permutation testing. The primary cutoff (20th/15th) is highlighted in red. Alternative cutoffs (10th/25th for TSC1-low; 10th/20th for high glial markers) yielded consistent significant co-localization (all p < 0.01), confirming the robustness of the spatial association.
